# Mechanism of Na-K-ATPase Inhibition by PGE2 in Intestinal Epithelial Cells

**DOI:** 10.3390/cells10040752

**Published:** 2021-03-29

**Authors:** Niraj Nepal, Subha Arthur, Jennifer Haynes, Balasubramanian Palaniappan, Uma Sundaram

**Affiliations:** Department of Clinical and Translational Sciences, Appalachian Clinical and Translational Science Institute, Joan C. Edwards School of Medicine, Marshall University, 1600 Medical Center Drive, Huntington, WV 25701, USA; nepal@marshall.edu (N.N.); arthursu@marshall.edu (S.A.); haynesje@marshall.edu (J.H.); palaniappan@marshall.edu (B.P.)

**Keywords:** PGE2, Intestinal epithelial cells, Villus, Na-K-ATPase

## Abstract

The primary means of intestinal absorption of nutrients by villus cells is via Na-dependent nutrient co-transporters located in the brush border membrane (BBM). These secondary active co-transport processes require a favorable transcellular Na gradient that is provided by Na-K-ATPase. In chronic enteritis, malabsorption of essential nutrients is partially due to inhibition of villus Na-K-ATPase activity mediated by specific immune inflammatory mediators that are known to be elevated in the inflamed mucosa. However, how Prostaglandin E2 (PGE2), a specific mediator of nutrient malabsorption in the villus BBM, may mediate the inhibition of Na-K-ATPase is not known. Therefore, this study aimed to determine the effect of PGE2 on Na-K-ATPase in villus cells and define its mechanism of action. In vitro, in IEC-18 cells, PGE2 treatment significantly reduced Na-K-ATPase activity, accompanied by a significant increase in the intracellular levels of cyclic Adenosine Monophosphate (cAMP). The treatment with cAMP analog 8-Bromo-cAMP mimicked the PGE2-mediated effect on Na-K-ATPase activity, while Rp-cAMP (PKA inhibitor) pretreatment reversed the same. The mechanism of inhibition of PGE2 was secondary to a transcriptional reduction in the Na-K-ATPase α1 and β1 subunit genes, which was reversed by the Rp-cAMP pretreatment. Thus, the PGE2-mediated activation of the PKA pathway mediates the transcriptional inhibition of Na-K-ATPase activity in vitro.

## 1. Introduction

Na-K-ATPase is an integral plasma membrane protein present in the basolateral membrane (BLM) of epithelial cells and transports 3 Na out of and 2 K into the cell by utilizing one ATP per transport cycle. As a consequence of pumping Na out of the cell, Na-K-ATPase generates a favorable transcellular Na gradient required for Na-dependent nutrient absorption in the brush border membrane (BBM) of the intestinal epithelial cells. Thus, Na-K-ATPase plays a vital role in driving transporters, specifically Na-dependent transporters present on the BBM of absorptive intestinal villus cells.

Three distinct subunits: alpha (α), beta (β), and gamma (γ) constitute a fully functional Na-K-ATPase enzyme. Of these subunits, the α and β subunits are crucial for the proper functioning of Na-K-ATPase, whereas the γ subunit is not essential for its function [1,2,3]. The catalytic α subunit that does the pumping function can be found in four different isoforms (α1, α2, α3, and α4) and is reported to be expressed in a tissue-specific manner [1,3,4]. However, the regulatory β subunit facilitates maturation of the α subunit by forming an α/β heterodimer and transporting this enzyme to the plasma membrane [2]. Of these isoforms α1 and β1 are ubiquitously present in intestinal mucosa [5,6]. The activity of Na-K-ATPase can be regulated by various mechanisms: (1) trafficking of pump from cytoplasm to the plasma membrane [7], (2) transcriptional regulation of subunits [8,9], and (3) phosphorylation and dephosphorylation of subunits [10,11,12].

Inflammatory bowel disease (IBD) is characterized by the malabsorption of nutrients and electrolytes, resulting in severe weight loss and malnutrition. Previous studies have shown that Na nutrient co-transport pathways in the BBM—namely, Na-glucose (SGLT1), Na-alanine (ATB0), Na-glutamine (B0AT1), Na-taurocholate (ASBT), and Na-adenosine (DMT1) co-transporters, which depend on the BLM Na-K-ATPase for their optimal activities—are inhibited in villus cells in a rabbit model of IBD [13,14,15,16]. In these cases, it has been reported that there is decreased Na-K-ATPase activity in villus cells, which implies that Na-K-ATPase might be at least in part responsible for the improper functioning of those transporters, thus leading to malabsorption of nutrients.

It has been well-established that, during IBD, various immune inflammatory mediators are produced endogenously in the intestine [17]. These immune inflammatory mediators work solely or synergistically with one another to affect electrolyte and nutrient transport pathways [18,19,20]. Immune inflammatory mediators such as arachidonic acid metabolites (AAMs), including prostaglandins (PGs) and leukotrienes (LTs), are prominently seen in the mucosa of patients with IBD. AAMs like PGs are formed through the cyclooxygenase (COX) pathway [21], whereas LTs are produced through the lipoxygenase (LOX) pathway [22]. The COX pathway derivative PGE2 was found to be responsible for the reduced activity of Na-dependent glutamine transporter B0AT1 and Na-K-ATPase in villus cells of IBD-induced rabbits [23]. More specifically, PGE2 reduced the B0AT1 activity by reducing its protein expression in the brush border membrane without affecting its affinity for its substrate glutamine. PGE2 has also been shown to affect numerous biological activities like water and electrolyte transport in the gut, have vasoactive effects, and induce smooth muscle contraction [14,24]. However, the specific molecular mechanism responsible for PGE2-mediated reduction of Na-K-ATPase activity in villus cells is yet to be determined. Therefore, this work aims to understand the molecular mechanism(s) involved in PGE2-mediated reduction of Na-K-ATPase in intestinal epithelial cells.

## 2. Materials and Methods

### 2.1. Reagents

All reagents were purchased from Cayman chemicals (Ann Arbor, MI, USA): Prostaglandin E2 (PGE2, Cat# 14010), AH6809 (Cat# 14050), Rp-cAMP (Cat# 16985), and 8-bromo-Cyclic AMP (Cat# 14431) were used in the experiments. All reagents were dissolved in DMSO to make the stock solutions. Final working concentrations of solutions contained less than 0.5% (*v*/*v*) DMSO. To check the effect of DMSO alone, an equal amount of DMSO was added in control experiments (vehicle control). Toxicity of all the drugs were assessed, and the lowest effective and safe dose was used for further experiments (Figure S1).

### 2.2. Cell Culture

The cells used in experiments were rat intestinal epithelial cells (IEC-18, American Type Culture Collection) between passages 5 and 20. IEC-18 cells are a non-transformed, polarized rat epithelial cell line that maintains its integrity and biochemical properties that are similar to in vivo mammalian intestinal epithelial cells. IEC-18 cells were grown in Dulbecco’s modified Eagle’s medium (DMEM), supplemented with 10% (*v*/*v*) fetal bovine serum, 100-U/L human insulin, 0.25 mM β-hydroxybutyric acid, and 100 units/mL penicillin and streptomycin. These cells were cultured in a humidified atmosphere of 10% CO_2_ at 37 °C. Cells were fed with fresh DMEM every other day. When the cells reached 100% confluence, it was considered as 0 day, and cells were grown until 4 days post-confluence. All of the experiments were conducted on day 4 post-confluence when they exhibit villus-like features of the small intestine [25].

### 2.3. Cell Viability Assays

For assessing the cell viability, the MTT (3-(4,5-dimethylthaizol-2-yl)-2,5-diphenlytetrazolium bromide) assay was performed following Vybrant MTT cell proliferation kit’s manual (Cat# V-13154, Thermo Fisher Scientific, Waltham, MA, USA). To perform the MTT assay, cells were seeded with an equal number of cells in a 96-well plate and cultured, then treated with test chemicals for the desired time. Once the cells were plated, they were grown until 4 days post-confluence. The medium was then removed from the wells and replaced with 100 μL of fresh medium. After that, 10 μL of 12 mM MTT solution (prepared in PBS) was added and incubated for at 37°C for 2 h. The whole medium was aspirated and replaced with 100 μL of SDS-HCl solution to each well and mixed thoroughly. The cells were incubated in SDS-HCl solution overnight (~12 h) at 37°C. Then, the solution was again mixed, and the absorbance was read on the spectrophotometer at 570 nm. Trypan blue exclusion assay was also performed to measure the cell viability.

### 2.4. Crude Plasma Membrane Preparation

Crude plasma membrane was prepared from IEC-18 cells following the method of Havrankova et al. [26]. Briefly, 100 mg of cell pellets were well mixed with 2.5-fold 0.001 M NaHCO_3_ (pH 7.4) prepared with a protease inhibitor cocktail (cOmplete^TM^, Cat# 11836153001, Millipore Sigma, St. Louis, MO, USA). Then, the mixture was homogenized 3 times (10 s each) with a homogenizer (IKA, Cat# 823707, T25 S1, Staufen, Germany). These homogenized cells were centrifuged at 600× *g* for 30 min. The resultant supernatant was centrifuged for 30 min at 20,000× *g*. The membrane was washed twice with 0.001 M NaHCO_3_. The final pellet was resuspended in 0.04 M Tris-HCl buffer (pH 7.4) containing the protease inhibitor cocktail. All procedures were carried out at 4 °C.

### 2.5. Na-K-ATPase Activity Assay

Na-K-ATPase activity was measured as Pi (Inorganic phosphate) liberated in plasma membrane fractions of villus cells according to protocol of Forbush et.al. [27]. Briefly, a solution I (Tris HCL (pH 7.4), MgSO4 (0.1 M), KCl (0.1 M), NaCl (0.1 M)) was first prepared with or without ouabain (Na-K-ATPase inhibitor). Then, 20 µg of plasma membrane preparation was added to solution I and incubated for 5 min at 37 °C. Subsequently, adenosine triphosphate (ATP; 2 mM) was added to solution I with the plasma membrane preparation and incubated for another 15 min at 37 °C. Then, solution II (ascorbic acid 0.49 M, 1 N HCl, 20% SDS and 10% ammonium molybdate) was added, followed by incubation for another 10 min in the ice-water bath. The reaction was stopped by the addition of solution III (2% arsenite, 2% sodium citrate and 2% acetic acid), followed by incubation for 10 min at 37 °C. Finally, the solution was read at 705 nm in a spectrophotometer. Enzyme-specific activity was expressed as nanomoles of Pi released per milligram protein per minute.

### 2.6. ^86^Rb^+^ Uptake for Na-K-ATPase Activity

For uptake studies, IEC-18 cells were grown on Transwell inserts (insert size 24 mm, pore size 0.4 µm; Corning, Cat# 29442, NY, USA) in 24-well plates. IEC-18 cells were plated with equal numbers of cells. Uptake studies for Na-K-ATPase were done using radioactive Rubidium (^86^Rb^+^, PerkinElmer, Waltham, MA, USA). ^86^Rb^+^ is comparable to K^+^ in chemical characteristics and has similar affinity for the Na-K-ATPase and, hence, was used to determine Na-K-ATPase activity [28,29]. Cells were incubated for 1 h in serum-free DMEM (SFM). The cells were subsequently washed with SFM and incubated for 10 min at 37 °C in SFM containing 20 μM monensin on both sides of the membrane. Then, cells were washed with SFM. Na-K-ATPase uptake studies were then performed by incubating cells for 15 min with reaction mixture (SFM and ^86^Rb^+^ (~1 μCi/well)) on the basolateral side of the membrane in the presence and absence of ouabain (1 mM). The reaction was stopped by the addition of ice-cold MgCl_2_ (10 mM), subsequently washed three times with MgCl_2_, and the cells were lysed with 800 μL of 1 N NaOH and incubation for 30 min at 70 °C, which was then mixed with 5 mL of Ecoscint A (National diagnostics). The vials were kept in darkness overnight, and the radioactivity retained by the cells was determined in a Beckman Coulter 6500 scintillation counter.

### 2.7. RNA Isolation and Quantitative Real-Time Polymerase Chain Reaction (qRT-PCR)

RNA was isolated from different experimental groups by using RNeasy mini kit obtained from Qiagen. qRT-PCR was performed using isolated total RNA by a two-step method. First, total RNA was used to synthesize cDNA using SuperScript III from (Invitrogen, Life Technologies) and an equal mixture of oligo (dT) primer and random hexamers. Second, newly synthesized cDNA was used as a template to perform real-time PCR using TaqMan Universal PCR master mix from Applied Biosystems (Foster city, CA, USA) according to manufacturer’s protocol. Rat-specific Na-K-ATPase α1- and β1-specific primers and probe were used for the qRT-PCR studies. Rat-specific β-actin primer and probe was used as a housekeeping gene to normalize the expression of samples.

### 2.8. cAMP Measurement

cAMP measurement was performed using the cAMP Direct immunoassay kit (Cat# ab65355, Abcam, Cambridge, MA, USA). Equal number of cells were seeded in a 35-cm^2^ dish and cultured until day 4 post-confluent, then treated with test chemicals for the desired time. The medium was aspirated followed by addition of 282 μL of 0.1 M HCl. The suspension was homogenized by pipetting up and down several times. Then, the suspension was centrifuged for 10 min at 14,000 rpm. The resultant supernatant was collected and used for further experiments. Next, the sample was diluted and mixed with acetylating reagents. After that, the samples were loaded in 96-well plates and incubated with cAMP antibody for an hour. cAMP-HRP was added to each well and incubated for another hour and washed 3 times with wash buffer. Subsequently, HRP developer was added and incubated for 1 h. The reaction was stopped by 1 M HCl, and the color developed was read at OD 450 nm using a plate reader (Spectramax i3x, Molecular Devices, San Jose, CA, USA).

### 2.9. Western Blot Analysis

Western blot analysis was performed on plasma membrane fractions prepared from different samples as described above. Equal amounts of protein (10 μg) were denatured in a sample buffer (10 mM Tris-HCl, pH 7, 12% glycerol, 2% SDS, 0.01% bromophenol blue and freshly added 1-mM dithiothreitol) and separated by electrophoresis on an 8% polyacrylamide gel. Proteins on the gel were transferred to a polyvinylidene fluoride (PVDF) membrane that was blocked with 5% milk or BSA in TBS (20 mM Tris, pH 7.5, and 150 mM NaCl) with 0.1% Tween-20 and then incubated with primary antibody against Na-K-ATPase α1 (Cat# 05-369, Millipore sigma, St.Louis, MO, USA) or Na-K-ATPase β1 (Cat# ab2873, Abcam, Cambridge, MA, USA) overnight at 4°C. Membranes were washed three times each with TBS and TBST, followed by incubation with secondary antibody for 1 h. Membranes were washed again three times each with TBS and TBST. ECL Western blotting detection reagent (GE healthcare Bio-Sciences, Piscataway, NJ, USA) was used to detect the immobilized protein. The chemiluminescence was detected using FluorChem instrument (Alpha Innotech, San Leandro, CA, USA) and analyzed with its software. Ezrin (Cat# ab4069, Abcam, Cambridge, MA, USA) antibody was used to normalize the expression levels of proteins in plasma membrane fractions.

### 2.10. Immunocytochemistry (ICC) Staining

IEC-18 cells were grown on a coverslip to day 4 post-confluence. Cells were treated with PGE2 or Rp-cAMP, as mentioned before. Following the treatment for 24 h, cells were fixed with 100% methanol (chilled at −20 °C) at room temperature for 5 min. After cells were permeabilized with PBST (PBS + 0.5% Tween 20) for 10 min, and cells were blocked with 3% BSA in PBST for 30 min. Then, the cells were incubated for an hour at room temperature with primary antibodies ZO-1 (Anti-Rabbit; Cat# 40-2200, Invitrogen Life technologies, Carlsbad, CA, USA) and Na-K-ATPase (Anti-mouse; Cat# 05-369, Millipore sigma, St. Louis, MO, USA). Alexa Fluor secondary antibodies (Invitrogen Life technologies, Carlsbad, CA, USA) were added, and the cells were incubated at room temperature for an hour. Cells were mounted with DAPI-containing mounting medium (Abcam PLC, Cambridge, MA, USA) and sealed with nail polish to prevent cells from drying. An EVOS microscope (Invitrogen Life technologies, Carlsbad, CA, USA) was used to capture images, and ImageJ software was used for analysis.

### 2.11. Protein Determination

Total protein was measured by the Lowry method using the DC Protein Assay Kit (Bio-Rad, Hercules, CA, USA). Different concentrations of BSA were used as standards. Samples were diluted to 200 µL with water. The diluted sample was mixed with 250 µL of DC Protein Assay reagent A (Cat# 500-0113, BIO-RAD, Hercules, CA, USA) and incubated for 2 min. Subsequently, the sample was incubated for another 15 min after the addition of 2 mL DC Protein Assay reagent B (Cat# 500-0114, BIO-RAD, Hercules, CA, USA). Finally, the sample was read at OD 750 nm using a spectrophotometer.

### 2.12. Statistical Analysis

All data presented had at least *n* = 4 per group of experiments, repeated with cells from different passages. The values were presented as the mean ± SEM, and *p*-values of < 0.05 were taken to indicate statistical significance. All of the data were analyzed using *t*-test, one-way (Dunnett’s multiple comparisons) or two-way analysis of variance (ANOVA, Tukey’s multiple comparison) using GraphPad Prism 7 software (San Diego, CA, USA).

## 3. Results

### 3.1. Effect of PGE2 on Na-K-ATPase in IEC-18 Cells

**Figure 1:**
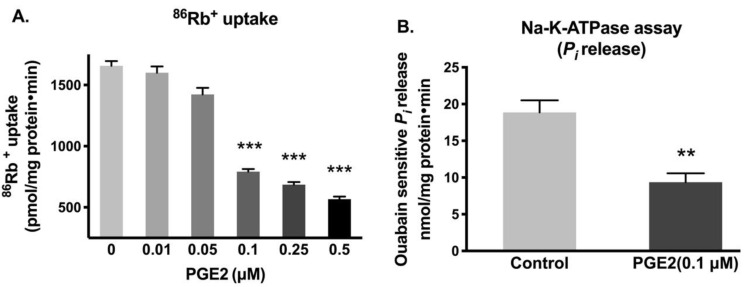
Effect of PGE2 exposure for 24 h on Na-K-ATPase activity in IEC-18 cells. (**A**). Measurement of Na-K-ATPase activity by ^86^Rb^+^ uptake (*n* = 6, Dunnett’s multiple comparison). (**B**). Measurement of Na-K-ATPase activity by *P_i_* release in plasma membrane preparations (*n* = 4, *t*-test). Values are represented as means ± SEM. ** *p* < 0.01 and *** *p* < 0.001 vs. 0 µM or control.

IEC-18 cells were treated with various concentrations of PGE2 for 24 h (two treatments 12 h apart), and the activity of Na-K-ATPase was measured by ^86^Rb^+^ uptake. The lowest dose of PGE2, which significantly diminished the Na-K-ATPase activity, was 0.1 µM (0.1 µM, 792 ± 53.2 vs. Control, 1656 ± 95.6 picomole/mg protein/min) (Figure 1A). The reduction of Na-K-ATPase activity in the plasma membrane by 0.1 µM of PGE2 was further corroborated by the inorganic phosphate (P_i_) release assay (Control, 18.9 ± 1.64 and PGE2 (0.1 µM), 9.36 ± 1.22 nanomole/mg protein/min; Figure 1B).

### 3.2. Effect of PGE2 on Cell Viability

**Figure 2:**
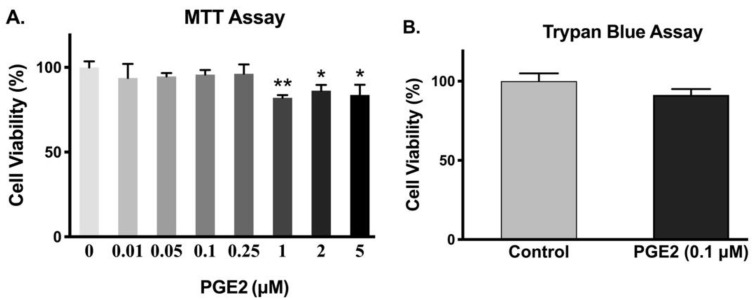
Effect of PGE2 exposure for 24 h on cell viability. (**A**). Measurement of cell viability by MTT assay (*n* = 6, Dunnett’s multiple comparison). (**B**). Measurement of cell viability by trypan blue assay (*n* = 4, *t*-test). Values are represented as means ± SEM; * *p* < 0.05 and ** *p* < 0.01 vs. 0 µM.

To ensure that a given dose of PGE2 did not affect the viability of IEC-18 cells, MTT and trypan blue assays were performed. Cell viability was not significantly altered with 0.25 µM or less concentration of PGE2 (Figure 2A). However, there was decreased cell viability in IEC-18 cells with 1 µM or higher concentration of PGE2. Therefore, the concentration we used for our experiments (0.1 µM) did not cause any cell death, which was also validated with the trypan blue exclusion assay (Figure 2B).

### 3.3. Prostaglandin Receptor Antagonist Blocked PGE2 Effect on Na-K-ATPase

**Figure 3:**
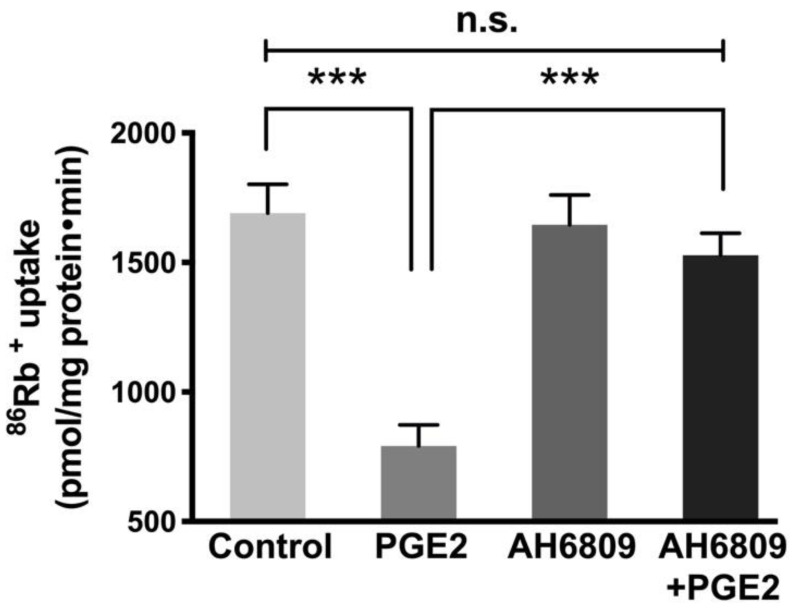
Effect of PGE2 (0.1 µM) receptor inhibitor AH6809 (5 µM) on Na-K-ATPase activity in IEC-18 cells. Measurement of Na-K-ATPase activity by ^86^Rb^+^ uptake. Values are represented as means ± SEM (*n* = 6, Tukey’s multiple comparison). *** *p* < 0.001; n.s., not significant.

The PGE2 receptor antagonist AH6809 was used to determine if the PGE2 effect is specifically mediated through its receptor activation in IEC-18 cells. AH6809 is an EP and DP receptor antagonist that blocks EP1, EP2, EP3, EP4 and DP1 receptors present in cells [30]. Previous studies from our laboratory have shown that EP2 and EP4 receptors are present in IEC-18 cells (data not shown). AH6809 (5 μM) blocks the PGE2 signaling in various cells [31]. Therefore, in this study, IEC-18 cells were pretreated with 5 μM of AH6809 for an hour, followed by PGE2 treatment for 24 h (two treatments 12 h apart). As shown in Figure 3, PGE2 significantly inhibited Na-K-ATPase, however, pretreatment with AH6089 followed by PGE2 treatment, it prevented the inhibition produced by PGE2 (Control, 1690 ± 111.1, PGE2, 792 ± 81.1, AH6089, 1645 ± 115 and AH6089 + PGE2, 1528 ± 85.4 picomole/mg protein/min). This indicates that the inhibitory effect of PGE2 is mediated through its receptors EP2 and/or EP4 in IEC-18 cells.

### 3.4. Effect of PGE2 on Intracellular cAMP

**Figure 4:**
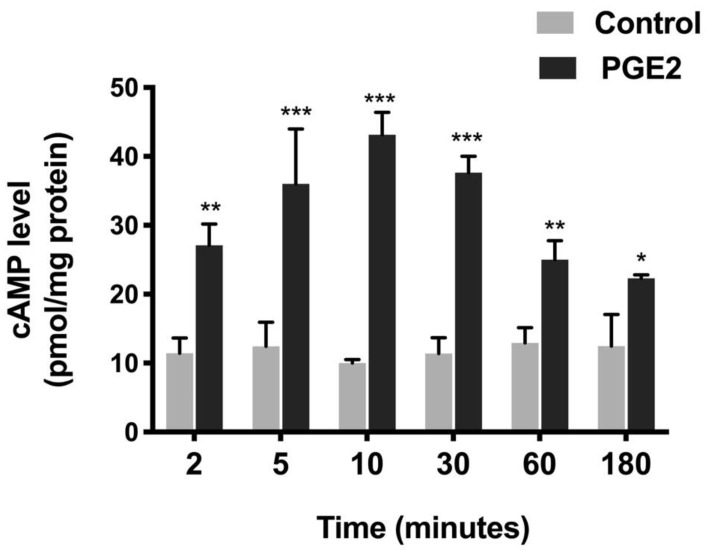
Level of cAMP at different time points during PGE2 (0.1 µM) treatment. Values are represented as means ± SEM (*n* = 8, *t*-test). * *p* < 0.05, ** *p* < 0.01, and *** *p* < 0.001 vs. Control. Levels of cAMP in controls did not change significantly at different time points.

In many systems, PGE2 is known to mediate its action via the second messenger cAMP, which is involved in various physiological and pathophysiological processes [32]. Thus, the measurement of cAMP was conducted. PGE2 treatment increased the cAMP levels in IEC-18 cells, as shown in Figure 4. cAMP increased 2.4-fold (Control, 11.4 ± 2.3 and PGE2, 27.09 ± 3.1 picomole/mg protein) within two min and peaked at 10 min (4.3-fold, Control, 10.1 ± 0.5 and PGE2, 43.2 ± 3.3 picomole/mg protein) with PGE2 treatment. The levels reduced gradually at later time points (30 min, 3.3-fold; Control, 11.4 ± 2.9 and PGE2, 37.7 ± 2.4, 60 min, 1.9-fold; Control, 12.9 ± 2.2 and PGE2, 25 ± 2.8, 180 min, 1.8-fold; and Control, 12.5 ± 4.6 and PGE2, 22.3 ± 0.6 picomole/mg protein) but were still significantly higher than the control. Thus, these data indicated that PGE2 increased the cAMP levels in IEC-18.

### 3.5. Effect of an Analog of cAMP on Na-K-ATPase Activity in IEC-18 Cells

**Figure 5:**
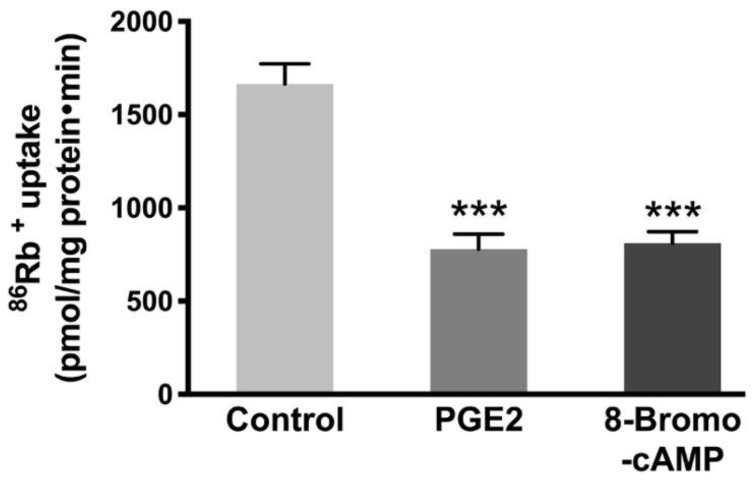
Effect of cAMP analog 8-Bromo-cAMP (0.1 mM) on the Na-K-ATPase activity in IEC-18 cells. Measurement of Na-K-ATPase activity by ^86^Rb^+^ uptake. Values are represented as means ± SEM (*n* = 6, Tukey’s multiple comparison). *** *p* < 0.001 vs. Control.

To know whether increased cAMP is responsible for the reduction of Na-K-ATPase, we used an analog of cAMP, 8-Bromo-cAMP. Previous studies have shown that 8-Bromo-cAMP (0.1 mM) activates cyclic-AMP dependent protein kinases in epithelial cells [33]. Therefore, IEC-18 cells were treated with 0.1 mM 8-Bromo-cAMP for 24 h (two treatments 12 h apart) followed by ^86^Rb^+^ uptake. The Na-K-ATPase activity was found to be diminished with the 8-Bromo-cAMP treatment comparable to PGE2 (Figure 5, Control, 1665 ± 108.4, PGE2, 780 ± 79.6, and 8-Bromo-cAMP, 779.8 ± 60.5 picomole/mg protein/min).

### 3.6. Effect of PKA Pathway Inhibition on Na-K-ATPase Activity in IEC-18 Cells

**Figure 6:**
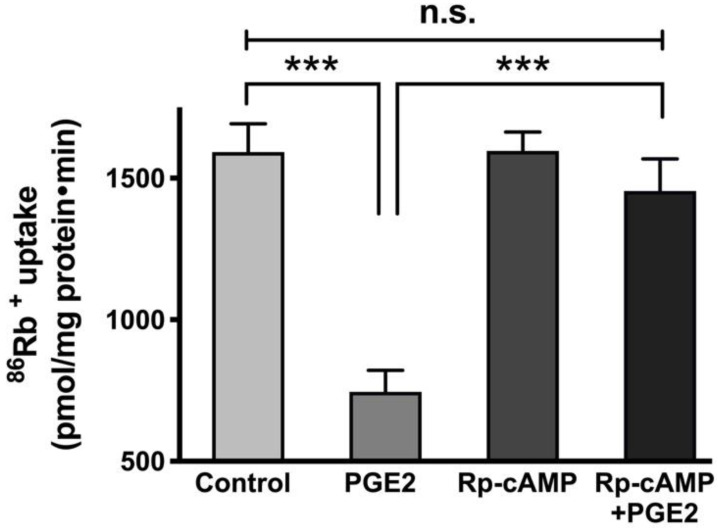
Effect of PGE2 (0.1 µM) and PKA inhibitor Rp-cAMP (10 µM) on the Na-K-ATPase activity in IEC-18 cells. Measurement of Na-K-ATPase activity by ^86^Rb^+^ uptake. Values are represented as means ± SEM (*n* = 6, Tukey’s multiple comparison). *** *p* < 0.001; n.s., not significant.

It has been demonstrated that cAMP mediates its effects via the activation of protein kinase A (PKA) and subsequent phosphorylation of many proteins [34]. To see whether PKA plays an active role in the regulation of Na-K-ATPase by PGE2, cells were pretreated with 10 μM of the PKA inhibitor Rp-cAMP (IC_50_ = 12.5 μM [35,36]) for an hour. After pretreatment, cells were treated with PGE2 for 24 h (two treatments 12 h apart), and cellular uptakes for ^86^Rb^+^ were performed. Pretreatment with Rp-cAMP prevented the PGE2-mediated reduction of Na-K-ATPase (Control, 1592 ± 100.9, PGE2, 746 ± 75.3, Rp-cAMP, 1596 ± 67.3, and Rp-cAMP + PGE2, 1455 ± 113.5 picomole/mg protein/min; Figure 6).

### 3.7. Na-K-ATPase α1 and β1 Subunit mRNA Abundance after PGE2 Treatment

**Figure 7:**
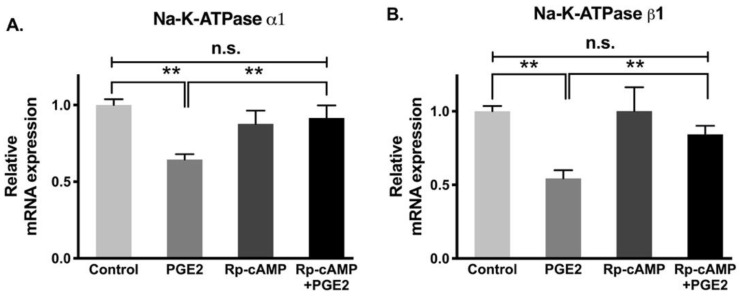
qRT-PCR analysis of IEC-18 cells with PGE2 (0.1 µM) and Rp-cAMP (PKA inhibitor, 10 µM). Values are relative to the control and normalized to β-actin. (**A**). Na-K-ATPase α1. (**B**). Na-K-ATPase β1. Values are represented as mean ± SEM (*n* = 4, Tukey’s multiple comparison). ** *p* < 0.01; n.s., not significant.

Gene and protein expression levels of Na-K-ATPase subunits, specifically α and β, are correlated with its functional activity. While the α subunit is accountable for Na-K-ATPase pumping activity, the β subunit does not contribute any pumping activity directly but instead supports the α subunit by proper folding and transporting α subunit from the cytoplasm to the plasma membrane. Therefore, to determine whether changes in Na-K-ATPase activity mediated by PGE2 are transcriptionally regulated, we performed qRT-PCR on both Na-K-ATPase α1 and β1 subunits. Na-K-ATPase α1 subunit mRNA was significantly decreased when exposed to PGE2, while Rp-cAMP pretreatment prevented the reduction of the Na-K-ATPase α1 subunit (Figure 7A, control, 1.00 ± 0.04, PGE2, 0.65 ± 0.04, Rp-cAMP, 0.88 ± 0.13, and PGE2 + Rp-cAMP, 0.93 ± 0.09). Similarly, there was a decrease in the Na-K-ATPase β1 subunit levels when treated with PGE2, but the reduction was partially abolished when pretreated with Rp-cAMP (Figure 7B, control, 1.00 ± 0.05, PGE2, 0.55 ± 0.06, Rp-cAMP, 1.01 ± 0.16, and PGE2 + Rp-cAMP, 0.85 ± 0.05). These data indicate that the Na-K-ATPase α1 and β1 subunits were transcriptionally downregulated when treated with PGE2. Additionally, the PGE2-mediated decrease in the Na-K-ATPase α1 and β1 subunit mRNA abundances was prevented with Rp-cAMP, indicating that the PKA pathway is responsible for the PGE2-mediated regulation of Na-K-ATPase.

### 3.8. Na-K-ATPase α1 and β1 Subunit Protein Expression after PGE2 Treatment

**Figure 8:**
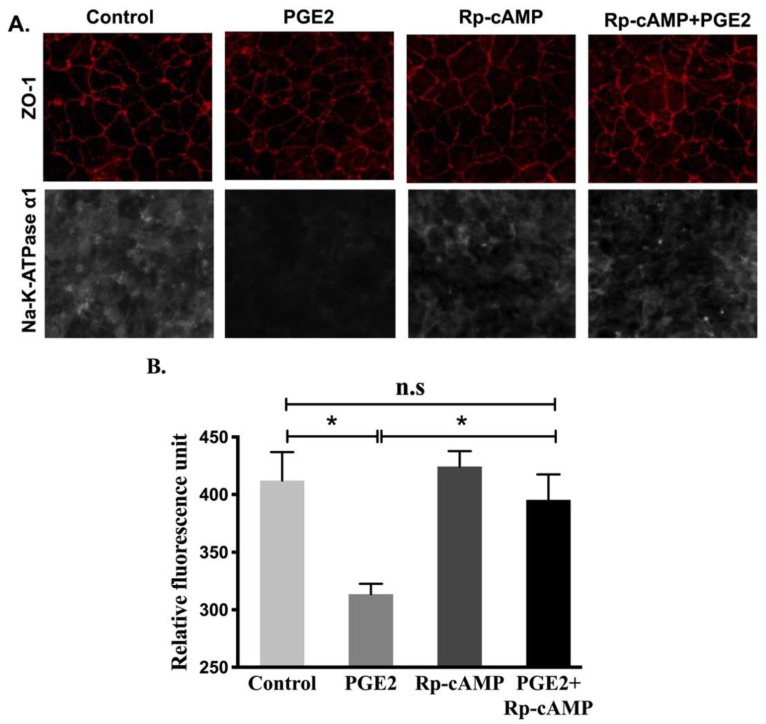
Immunocytochemistry of IEC-18 cells treated with PGE2 (0.1 µM) and Rp-cAMP (PKA inhibitor, 10 µM). (**A**). Representative images of Na-K-ATPase α1 (grey) and ZO-1 (red) (20X). (**B**). Quantification of Na-K-ATPase α1 fluorescence. Values are represented as mean ± SEM (*n* = 6, Tukey’s multiple comparison). * *p* < 0.01 vs. Control.

**Figure 9:**
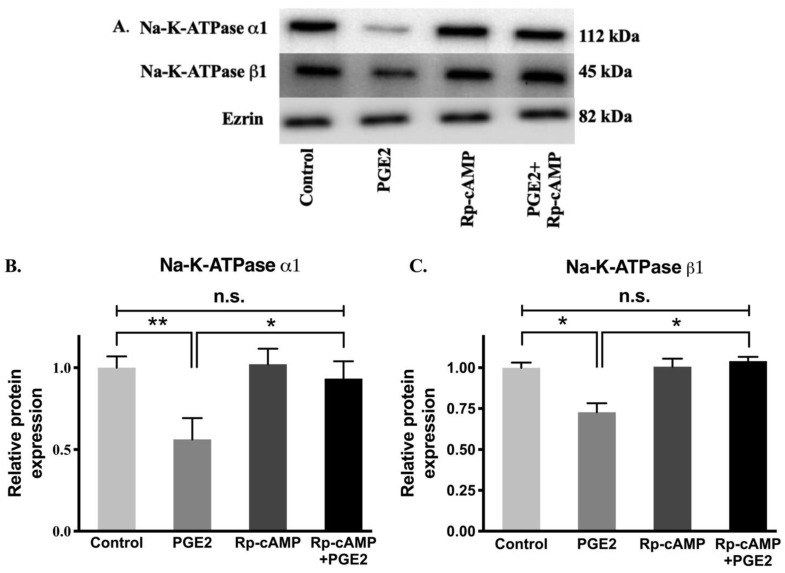
Western blot analysis of IEC-18 cells treated with PGE2 (0.1 µM) and Rp-cAMP (PKA inhibitor, 10 µM). (**A**). Representative western blot of Na-K-ATPase α1, Na-K-ATPase β1, and Ezrin (internal control). Densitometric quantitation of blots. (**B**) Na-K-ATPase α1 and (**C**) Na-K-ATPase β1. Values are relative to the control and normalized to Ezrin. Values are represented as mean ± SEM (*n* = 4, Tukey’s multiple comparison). * *p* < 0.05 and ** *p* < 0.01; n.s., not significant.

Protein levels of the Na-K-ATPase α1 and β1 subunits were determined by performing immunocytochemistry of cell monolayers and Western blots analysis on plasma membrane fractions for all experimental conditions. Immunocytochemistry (Figure 8) and Western blot analysis (Figure 9) showed that relative levels of Na-K-ATPase α1 protein expression were significantly lowered after PGE2 treatment, whereas this reduction was blocked when treated with Rp-cAMP (Figure 9A,B). Similarly, there was also a significant decrease in the Na-K-ATPase β1 subunit protein expression in plasma membrane fractions when treated with PGE2, and this was reversed when treated with Rp-cAMP (Figure 9A,C). Therefore, the expression of the Na-K-ATPase α1 and β1 subunits mRNA correlated with its protein expression in plasma membrane fractions. These data indicate that PGE2 regulates Na-K-ATPase transcriptionally through the cAMP-activated PKA pathway.

## 4. Discussion

Prostaglandins play an essential role in maintaining normal physiological processes such as intestinal secretion, motility, and mucosal protection [22]. However, they have also been seen to be increased in the mucosa during chronic enteritis [37,38]. PGE2 has been extensively studied in the various process such as apoptosis [39] and inflammatory processes [22]. With relevance to intestinal absorption, PGE2 has been shown to decrease active sodium and chloride absorption and increase chloride secretion in in vitro studies [37,38]. PGE2 has also been involved in the regulation of intestinal epithelial transporters, including Na-glucose (SGLT1) [40,41], Na/H exchanger [42,43], Na-K-ATPase [44], and Cl/HCO_3_ exchanger [45]. Moreover, our laboratory has also shown that the cyclooxygenase pathway, which produces prostaglandins, is involved in the reduction of Na-glutamine uptake (B0AT1) and Na-K-ATPase activity in villus cells in the inflamed intestines of rabbits [46]. Given this background, it is evident that PGE2 plays a crucial role in regulating intestinal epithelial cell absorptive pathways, including Na-K-ATPase during chronic enteritis. However, the molecular mechanism of regulation of Na-K-ATPase by PGE2 in intestinal epithelial cells during chronic intestinal inflammation is not known.

In the present study, we demonstrated that the inflammatory mediator PGE2 regulates Na-K-ATPase in the basolateral membrane, which, in turn, may partially be responsible for the regulation of several secondary active Na-dependent nutrient and electrolyte transporters in the brush border membrane of intestinal epithelial cells. This study also revealed that PGE2 reduces Na-K-ATPase activity through the activation of the PKA-mediated pathway in rat intestinal epithelial cells (IEC-18 cells), which physiologically behave like absorptive villus cells when grown to four days post-confluence [5,25].

PGE2 is a well-known regulator of Na-K-ATPase activity in numerous tissue types. A PGE2-mediated inhibitory effect in Na-K-ATPase has been seen in various organs, including the heart [47], liver [48,49], and kidneys [50]. Similar to these findings, our data also demonstrates that 0.1 μM of PGE2 for 24 h is optimal for the reduction of Na-K-ATPase activity in IEC-18 cells. Our qRT-PCR data showed that PGE2 transcriptionally reduces Na-K-ATPase α1 and β1, which led to a decrease in plasma membrane protein expression of both subunits, as demonstrated by the Western blot analysis. Immunofluorescence studies also confirmed that there was a significant decrease in the membrane expression of the functional Na-K-ATPase α1 subunit in cells treated with PGE2. However, as demonstrated by Western blot and immunofluorescence studies, Rp-cAMP pretreatment reversed the downregulation of Na-K-ATPase α1 subunit expression, indicating that the cAMP-mediated pathway was responsible for the inhibition of Na-K-ATPase activity in PGE2-treated cells. Further analysis of the data revealed that there was a significant correlation between Na-K-ATPase function and protein α1 subunit expression (R^2^ value 0.99; Pearson’s r correlation test).

Previously, our laboratory showed that IEC-18 cells have PGE2 receptors (EP2 and EP4). The specific inhibitor AH6809 is an EP and DP receptor antagonist and inhibits these receptors with equal affinity [30]. When IEC-18 cells were pretreated with AH6809, the reduction of Na-K-ATPase due to PGE2 treatment was prevented. These data demonstrate that the decrease of Na-K-ATPase activity is mediated through PGE2 receptors. These receptors mediate their action through secondary messengers such as Ca^2+^ or cAMP [51,52]. We observed that PGE2 increases the intracellular cAMP levels. Therefore, to demonstrate the role of cAMP in the reduction of Na-K-ATPase, we treated cells with a cAMP analog (8-Bromo-cAMP), which produced similar effects on Na-K-ATPase activity comparable to that by PGE2 treatment.

PGE2 has been shown to activate signaling pathways through PKA or PKC downstream of the second messenger cAMP or Ca^2+^ in various cell types [53,54]. PKA has been shown to have differential regulation (activation, inhibition, or no effect) of Na-K-ATPase based on the tissue types. The discrepancy in the regulation of Na-K-ATPase by PKA might be due to the phosphorylation of different isoforms of Na-K-ATPase subunits [7]. Oliveira et al. reported that the short-term treatment of PGE2 has a PKA-mediated inhibitory effect on Na-K-ATPase hippocampal tissue, likely due to phosphorylation of the Ser^943^ residue of the α subunit [55]. On the other hand, cAMP-mediated PKA has also been shown to regulate Na-K-ATPase transcriptionally. Dagenais et al. reported that the activation of PKA by a cAMP analog subsequently phosphorylated the transcription factor CREB and, eventually, increased the synthesis of mRNA of the α1 subunit but not the β1 subunit after eight h of treatment in isolated rat alveolar epithelial cells [56]. However, there was no significant change in the α1 mRNA level after 24 h of cAMP analog treatment. Additionally, three prostaglandin response elements (PGRE) were identified in the β1 subunit, which, in response to PGE1 (four h of treatment), increased the transcription of the β1subunit by activating transcription factors CRE and CREB [57,58,59]. Contrary to this finding, in our study, we found that cAMP-mediated PKA is responsible for the inhibition of Na-K-ATPase. The discrepancy in the results may be due to the different tissues of origin that we used, the concentration of the PGE2, and/or the exposure time. Another possible reason for this discrepancy might be that the observed effect is an early response. Moreover, in a study by Mony et al., in the hypoxic condition in cancer, transcription factors hypoxia inducing factor (HIF-1α) and Smad3 were shown to bind to the β1 subunit gene and repress the expression of β1 subunit [60]. Therefore, the prolonged treatment with PGE2, as seen in this study, might result in transcription repression of the β1 subunit by transcription factors like HIF-1α and Smad3.

## 5. Conclusions

**Figure 10:**
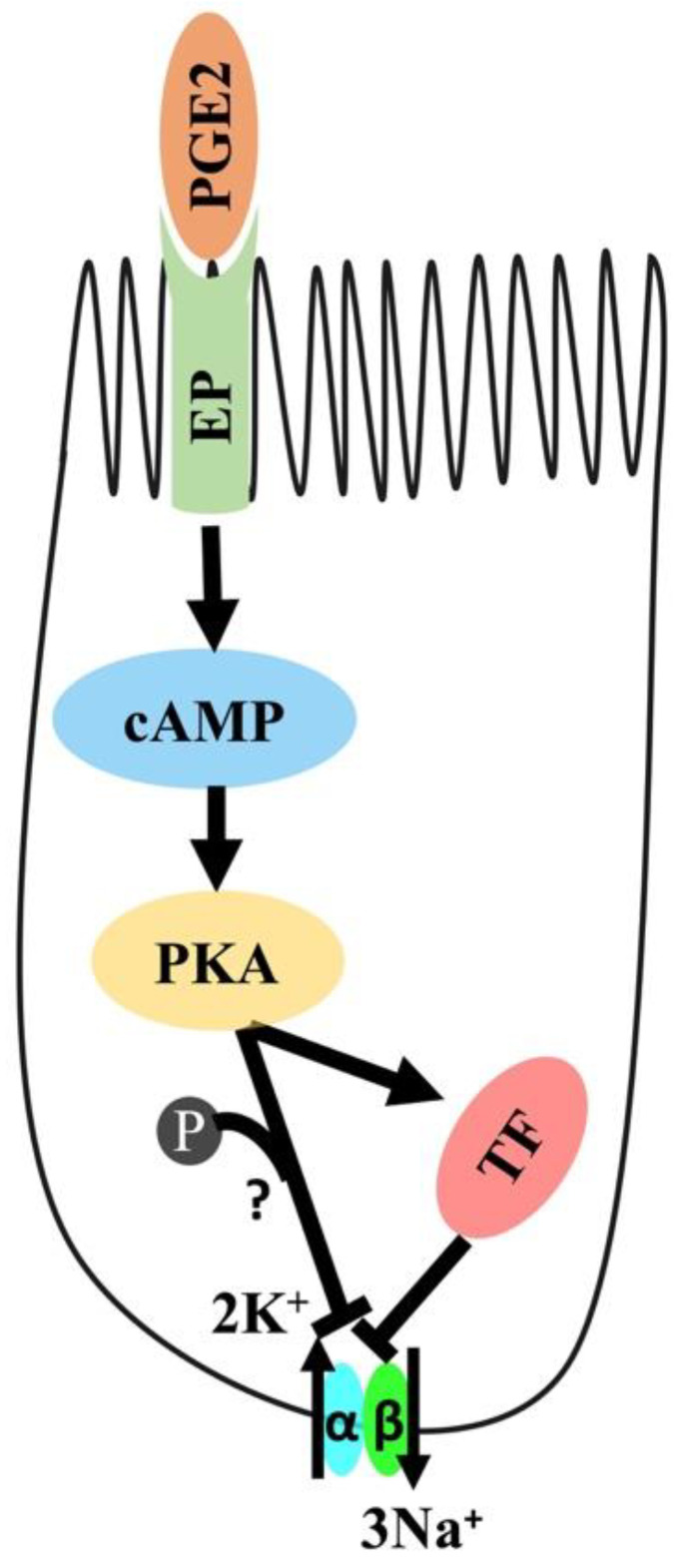
Proposed mechanism of PGE2-mediated regulation of Na-K-ATPase in intestinal epithelial cells. PGE2; Prostaglandin-E2, EP: Prostaglandin receptor, PKA: Protein Kinase A, and TF: Transcription Factor.

The current study indicates that PGE2 reduces Na-K-ATPase activity via the cAMP-mediated PKA pathway, as shown in Figure 10. The mechanism involves the binding of PGE2 to prostaglandin receptors, leading to increased intracellular cAMP production and the subsequent activation of the PKA pathway, which eventually leads to a decrease in the Na-K-ATPase activity. The reduction in activity is due to transcriptional repression of genes encoding the α1 and β1 subunits of Na-K-ATPase.

## Data Availability

Not applicable.

## Supplementary Materials

The following are available online at https://www.mdpi.com/article/10.3390/cells10040752/s1: Figure S1: Effect of different concentrations of reagent(drug) exposures for 24 h on cell viability. (A) AH6809. (B) Rp-cAMP. (C) 8-Bromo-cAMP. Measurement of cell viability by MTT assay. Values are relative to 0 μM and are represented as the means ± SEM, *n* = 6. * *p* < 0.05 vs. 0 μM.
