# Supplementary material for: Mechanism of Na-K-ATPase Inhibition by PGE2 in Intestinal Epithelial Cells
Source: Cells. 2021 Mar 29;10(4):752. doi: 10.3390/cells10040752 (PMC8066871; doi:10.3390/cells10040752)
Supplement: Supplementary file 1 [file cells-10-00752-s001.pdf]

Supplementary Materials:

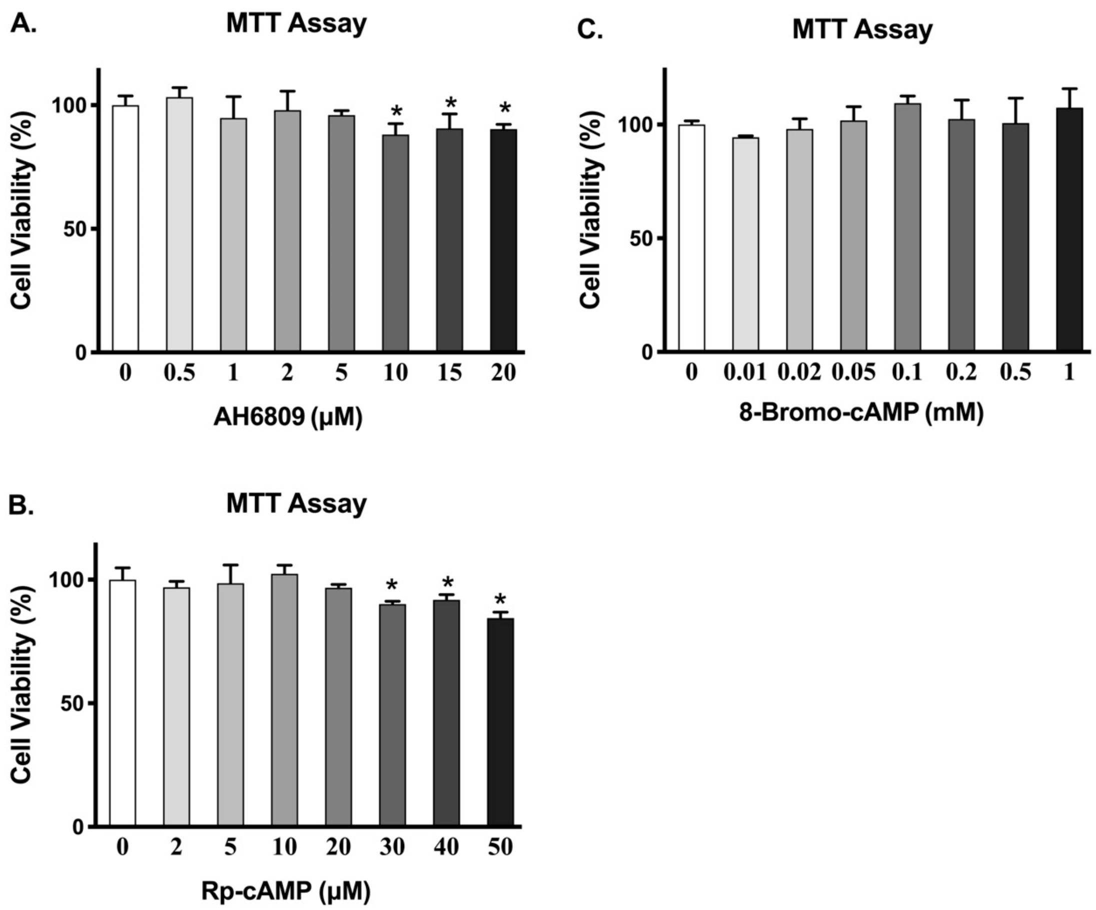

Figure S1. Effect of different concentration of reagents(drugs) exposure for 24 hours on cell viability. A. AH6809. B. Rp-cAMP. C. 8-Bromo-cAMP. Measurement of cell Viability by MTT assay. Values are relative to 0 μM and are represented as means ± SEM, n=6. *, $p < 0.05$ vs 0 μM.
